# Wnt-C59 arrests stemness and suppresses growth of nasopharyngeal carcinoma in mice by inhibiting the Wnt pathway in the tumor microenvironment

**DOI:** 10.18632/oncotarget.3982

**Published:** 2015-05-04

**Authors:** Yue Cheng, Yee Peng Phoon, Xiwan Jin, Shing Yee Steffi Chong, Joseph Chok Yan Ip, Bonnie Wing Yan Wong, Maria Li Lung

**Affiliations:** ^1^ Department of Clinical Oncology, Center for Nasopharyngeal Carcinoma Research, University of Hong Kong, Laboratory Block, Pokfulam, Hong Kong SAR, China

**Keywords:** Wnt-C59, nasopharyngeal carcinoma, cancer stem cells, Wnt pathway, tumor suppression

## Abstract

Wnt/β-catenin signaling is responsible for the generation of cancer stem cells (CSCs) in many human tumors, including nasopharyngeal carcinoma (NPC). Recent studies demonstrate that Wnt or PORCN inhibitor, Wnt-C59, inhibits tumor growth in MMTV-WNT1 transgenic mice. The effect of Wnt-C59 in human tumors is not clear. In this study, the NPC cell lines investigated manifest heterogeneous responses to Wnt-C59 treatment. Wnt-C59 decreased tumor growth of SUNE1 cells in mice immediately following the administration of Wnt-C59. Mice injected with HNE1 cells did not develop visible tumors after the treatment of Wnt-C59, while control mice developed 100% tumors. Wnt-C59 inhibited stemness properties of NPC cells in a dosage-dependent manner by arresting sphere formation in both HNE1 and SUNE1 cells. Thus, Wnt-C59 has the potential to eradicate CSCs in human tumors. Active β-catenin and Axin2 proteins were strongly expressed in stromal cells surrounding growing tumors, confirming the importance of Wnt signaling activities in the microenvironment being driving forces for cell growth. These novel findings confirm the ability of Wnt-C59 to suppress Wnt-driven undifferentiated cell growth in NPC. Both anti-Wnt signaling and anti-CSC approaches are feasible strategies in cancer therapy.

## INTRODUCTION

The central concept of cancer stem cells (CSCs) is that the bulk of tumor cells contain a tiny cell population having self-renewal ability to sustain the long-term tumor proliferation against chemotherapy. Efforts to control the growth of these unique tumor cells are a major effort in current translational and clinical cancer research [1, 2]. A growing number of studies indicate that inappropriate activation of *Wnt*/β*-catenin* signaling correlates with the generation of CSCs in many tumor types [3-8]. Therefore, targeting of this pathway may provide the means to safely eradicate CSCs and identify novel molecular targets for cancer therapy [9, 10].

Activation of *Wnt*/β*-catenin* signaling associated with self-renewal is context-dependent in many human tumors [11-13]. Dosage levels of *Wnt* signaling and genetic background of tissues play critical roles in regulating self-renewal tumor cell networks [3, 14, 15]. Lack of knowledge underlying signal transduction mechanisms clearly impedes the exploration of effective therapeutic targets for this pathway [9, 10, 16]. A few promising *Wnt* targeting molecules, such as ICG001 [17], XAV939 [18], and Wnt-C59 [19], have been recently reported. Porcupine (PORCN) is a membrane bound O-acyltransferase (MBOAT) family that is essential for Wnt palmitoylation, secretion, and other biological activities, indicating that PORCN activity is a key modulator of *Wnt* signaling [16, 20]. Wnt-C59 is a strong and specific *PORCN* inhibitor. This small molecule is similar to other *Wnt* inhibitors, IWP family and LGK974, that inactivate *PORCN* function by directly inhibiting the *PORCN* active site or affecting regulators such as *Axin2* [7, 16, 20, 21]. A model of fine-tuning of the *Wnt* pathway by Wnt-C59 was proposed in which either β*-catenin*-mediated or its downstream components-mediated activities are arrested by *PORCN* inhibitors [22]. Several recent studies provide evidence showing that *PORCN* inhibitors, including Wnt-C59 and LGK974, inhibit or delay tumor growth in mouse models. These experiments were performed in MMTV-WNT1 transgenic mice and findings indicate that mammalian physiological *Wnt* signaling is sensitive to *PORCN* expression levels. Small changes in PORCN activity can induce significant inhibitory effects in rodent tumor models. Notably, Wnt-C59 and LGK974 are safe agents as verified in these animal studies and the latter is now in the phase I clinical trials [20, 23].

Recent findings reveal the vital role of *Wnt* signaling in both epithelial and stromal cells; inhibitory effects of Wnt-C59 mimick the *PORCN* knockout mouse to block proliferation of intestinal stem cells at physiological conditions [24]. However, whether targeting the Wnt pathway can be achieved to eradicate human CSCs by affecting the tumor microenvironment is largely unknown. Using reduced expression of *Axin2* mRNA as a reporter, Liu et al screened many human tumor cells, including brain, lung, and colon cancers and lymphoma/leukemia, and only detected a few (31%, 31/96 cases) head and neck squamous cell carcinoma (HNSCC) cell lines that were sensitive to the *PORCN* inhibitor treatment. One of these HNSCC cell lines, HN30, delays tumor growth within a 15-day period *in vivo,* following the administration of LGK974 [20]. Only a fraction of human tumors are sensitive to *PORCN* inhibitors, although aberrant *Wnt* signaling is widely reported in many human tumors [25]. We already know that precise regulation of PORCN activity is required for physiologically relevant expression levels of *Wnt* signaling, which might fine-tune network activities over a large dynamic range [19]. Therefore, many human tumor cells may not have responded to *PORCN* treatment because of the inappropriate or overexpressed levels of Wnt pathway activities or the involvement of other interacting pathways induced by aberrant *Wnt* signalling and accumulated mutations in these cells. However, such tumor models with dominant activation of *Wnt* signalling are limited for current studies.

Our early studies show that self-renewal networks in nasopharyngeal carcinoma (NPC) stem-like cells are precisely regulated by dominant physiological levels of *Wnt*/β*-catenin* signaling [3]. The exogenous Wnt signaling in these cells, interacting with *p53*, *RB1*, *TGF-*β, epithelial-mesenchymal transition and other pathways, can induce extensive signaling cascades and regulate *LIFR*- and *IL6ST*-mediated cell self-renewal networks. More importantly, inhibition of *Wnt* signaling in these stem-like cells could effectively suppress cell growth and sphere formation. Given the key roles of *Wnt*/β*-catenin* signaling in NPC [26], it is an attractive therapeutic approach to test *PORCN* inhibitor in this unique human cancer. Using several NPC cell lines, we now demonstrate that a *PORCN* inhibitor is able to inhibit *Wnt* signaling activities, arrest stemness properties, and suppress tumor cell growth in animal assays. Although these results vary depending on the context of NPC cells, these novel findings demonstrate that inhibition of *Wnt* signaling is a promising therapeutic approach in certain human tumors.

## RESULTS

### Wnt-C59-sensitive cell lines in mice have stemness properties in cell culture

**Figure 1:**
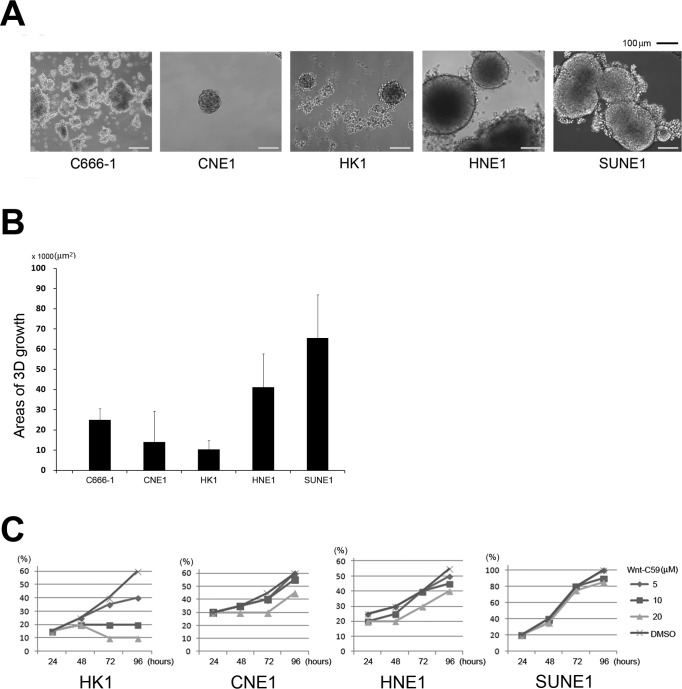
Stemness activity and Wnt-C59 treatment in NPC cells **A.** Phase-contrast sphere images from NPC cells. C666-1, CNE1, and HK1 cells generate relatively small spheres, but both HNE1 and SUNE1 cells can form large spheres (usually larger than 200 μm in diameter). **B.** Areas of average 3D growth are summarized. C666-1 cells grow more like colonies, but are counted as 3D growth. **C.** Cell proliferation influenced by concentrations of Wnt-C59 treatment. Wnt-C59 has a clear inhibitory effect on the growth of HK1 cells.

To determine whether NPC cells are sensitive to Wnt-C59 treatment, as reported in other head and neck tumors, we established a 3-dimensional (3D) cell culture system [27]. Plating cell densities influenced 3D sphere-formation abilities because most cells grew as a monolayer and only a small population of cells was capable of sphere formation. Cells growing as a monolayer surrounding the spheres might change to 3D growth, reflecting a NPC stemness property influenced by the local microenvironment. After 30 days culture, experiments were terminated and examined for sphere formation. C666-1 cells grow quickly, but most cells form a colony-like morphology and do not show obvious 3D cell growth. The sphere-formation ability was weak for both CNE1 and HK1 cells compared to HNE1 and SUNE1 cells that exhibited fast cell growth abilities and formed relatively large spheres (Figures 1A and 1B).

Previous studies indicated that Wnt-C59 did not significantly inhibit *in vitro* proliferation in 46 cancer cell lines at concentrations that might inhibit PORCN. The IC50 was greater than 50 μM in 87% of tested cell lines [23]. To investigate anti-stemness activities induced by Wnt-C59 in NPC cells, we investigated possible growth influences of this small molecule in a NPC cell line panel. As shown in Figure 1C, SUNE1 cells exhibited a strong tolerance to this agent, as reported in many other human cancer cell lines. Both CNE1 and HNE1 cells showed reduced growth abilities in the higher Wnt-C59 concentration (20 μM), while HK1 cells were sensitive to all concentrations (5 μM, 10 μM, and 20 μM) of Wnt-C59 treatments.

**Figure 2:**
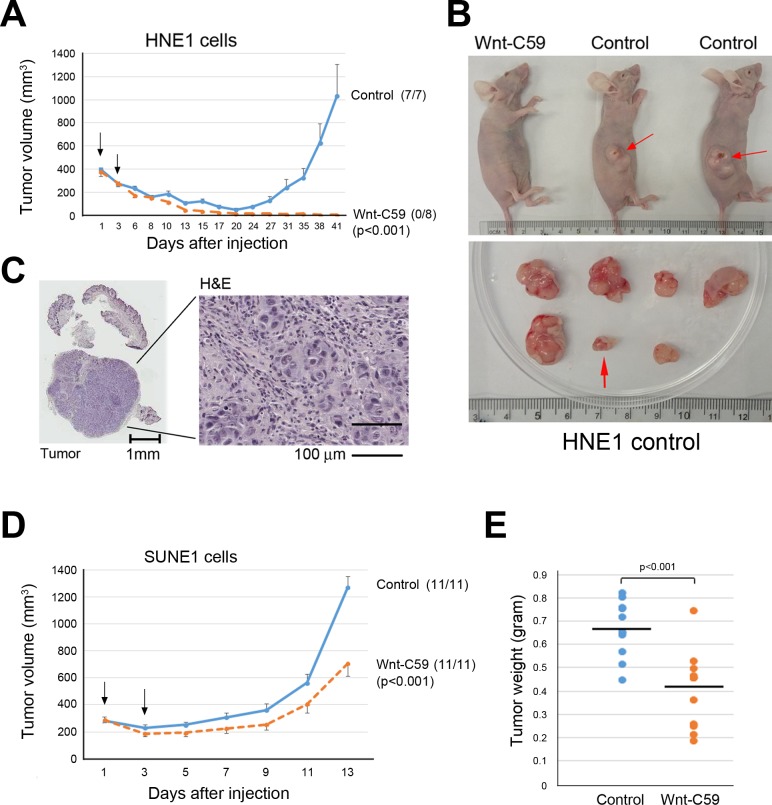
Wnt-C59 suppresses tumor growth in animals **A.** Growth curves show that Wnt-C59 completely suppresses the growth of *Wnt*-driven HNE1 cell in the animal assay. Arrowheads (on days 1 and 3 after cell injection) indicate the administration of Wnt-C59 by tail vein injection and drinking water, respectively. Tumor volume, mean ± SEM. No tumor growth is detected in Wnt-C59 treated animals, and the numbers in parentheses indicate the tumor incidence. **B.** Images of representative mice with or without tumors (top) and excised tumors from control group (bottom). Red arrowheads indicate growing tumors (top) and the smallest/longest latency period tumor (bottom) appearing on day 38 following injection of HNE1 cells in control mice. **C.** H&E staining shows the last tumor appearing on day 38 following injection of HNE1 cells in control mice. **D.** Growth curves show inhibitory effects of Wnt-C59 on SUNE1 cell growth in nude mice. Arrowheads (on days 1 and 3 after cell injection) indicate the administration of Wnt-C59 by tail vein injection and drinking, water, respectively. Tumor volume, mean ± SEM. **E.** Comparison of tumor weights in control and Wnt-C59-treated mice shows Wnt-C59 treatment clearly reduce tumor growth (*p* < 0.001).

Both SUNE1 and HNE1 cells showed a greater ability to form spheres (Figures 1A and 1B). Their IC50 for Wnt-C59 treatment at 48 hours is greater than 60 μM. Additionally, these two cell lines have obviously different *in vivo* cell growth dynamics, providing good animal models to determine biological effects of Wnt-C59 in NPC cells. HNE1 cells display less aggressive tumor growth in mice. While the majority of injected HNE1 cells (1 × 10^7^) die and injected cell nodules shrink for about 2 or 3 weeks post-injection, a fraction of the population survived and subsequently expanded to form progressively growing tumors after a period of latency, as seen in the control group mice (Figure 2A and 2B). In the 41-day testing period, the first and last growing tumors of the controls appeared on days 20 and 38, respectively, reflecting highly heterogeneous tumor behaviors. Shortly after the latency period, visible tumors were observed in all injected sites (7/7). In contrast, all injected tumor nodules shrank and disappeared following the administration of Wnt-C59 in 3 weeks and no visible and progressively growing tumors were detected in any injected sites (0/8) in 41 days, as shown in Figures 2A and 2B. Histological examination confirmed that all growing nodules in the control group contained tumor cells. Figure 2C shows a representative hematoxylin and eosin (H&E)-stained section of the smallest tumor finally appearing in mice. Therefore, the complete suppression of HNE1 cells *in vivo* confirms that the growth of the NPC subcutaneous tumor can be inhibited by Wnt-C59.

In contrast to HNE1, SUNE1 is a fast-growing cancer cell line. We tested tumor inhibitory effects of Wnt-C59 in nude mice following injection of SUNE1 cells. Figure 2D shows that Wnt-C59 inhibits SUNE1 cell growth immediately after administration of this agent for the 13-day period of tumor growth in mice. Compared to tumor sizes of control groups, average inhibited tumor sizes in Wnt-C59-treated animals were significantly smaller (*p* < 0.001). Comparison of tumor weights from both control and experimental groups is shown in Figure 2E, revealing that average tumor weights of the Wnt-C59-treated groups were significantly lower than for control mice (*p* < 0.001).

### Wnt-C59 suppresses *in vivo* tumor growth and high Wnt-C59 concentrations are also effective in *in vitro* assays

**Figure 3:**
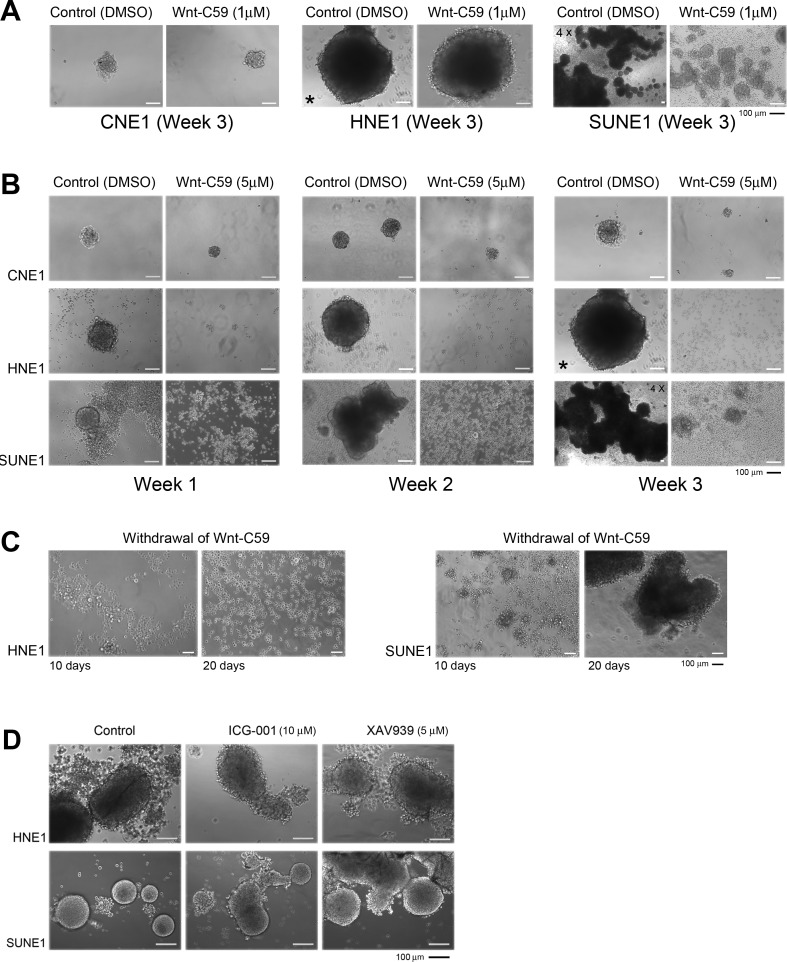
Wnt-C59 arrests 3D growth in NPC cells **A.** Phase-contrast images show that the Wnt-C59 (1 μM) clearly inhibits sphere formation in SUNE1 cells, compared with control cells. 4X, microscopy using the 4X objective, other images used 10X objective. * The same image is used for the HNE1 control cells on week 3 of Wnt-C59 treatment. **B.** The higher concentration of Wnt-C59 (5 μM) strongly inhibits sphere formation and growth in both HNE1 and SUNE1 cells, but not in CNE1 cells. Bar, 100 μm. **C.** Phase-contrast images of growing cells following the removal of Wnt-C59 treatment (5 μM). Only SUNE1 cells resume sphere growth recorded on days 10 and 20 and no sphere is detected in HNE1 cells. Bar, 100 μm. **D.** ICG001 and XAV939 do not cause obvious inhibition of sphere formation in both HNE1 and SUNE1 cells compared to their controls. Images are captured at the end of the first week of the treatments with these two agents. Bar, 100 μm.

A low concentration (1 μM) of Wnt-C59 treatment suppresses SUNE1 cells and 3D growth was clearly inhibited compared to control untreated cells (Figure 3A). The inhibitory effects of Wnt-C59 were dosage-dependent in these NPC cells; both 5 μM and 20 μM Wnt-C59 treatments can arrest sphere formation in both HNE1 and SUNE1 cell lines, but not CNE1 cells. However, cells showing a monolayer growth with Wnt-C59 treatments were continuously expanding and both 3D inhibition and monolayer growth were consistent, as observed from weeks 1 to 3 (Figure 3B).

After a three-week treatment, we withdrew Wnt-C59 and continued to culture cells for 10 to 20 days more under control cell culture conditions. No obvious 3D growth resumed or was detected in HNE1 cells, although cell densities and incubation periods were sufficient to generate 3D growth for untreated cells. However, monolayer-growing SUNE1 cells gradually formed huge spheres in 20 days under untreated culture conditions (Figure 3C). These findings indicated that inhibitory effects of Wnt-C59 on stemness were irreversible as seen in HNE1. The HNE1 cells with stemness properties in the whole cell populations could be safely eradicated by the *Wnt* inhibitor.

To verify the different effects of the Wnt inhibitors in our established sphere-formation system, we evaluated two other non-PORCN inhibitors, ICG001 and XAV939, as previously described. As expected, no obvious anti-stemness effects were observed by these two agents after three weeks of culture of HNE1 and SUNE1 cells (Figure 3D) even at higher concentrations of agents. Compared to control cells, no obvious growth inhibition was induced, confirming that these two *Wnt* inhibitors have different regulatory effects in the tumor cells.

### Wnt-C59 inhibits the Wnt pathway *in vivo*

**Figure 4:**
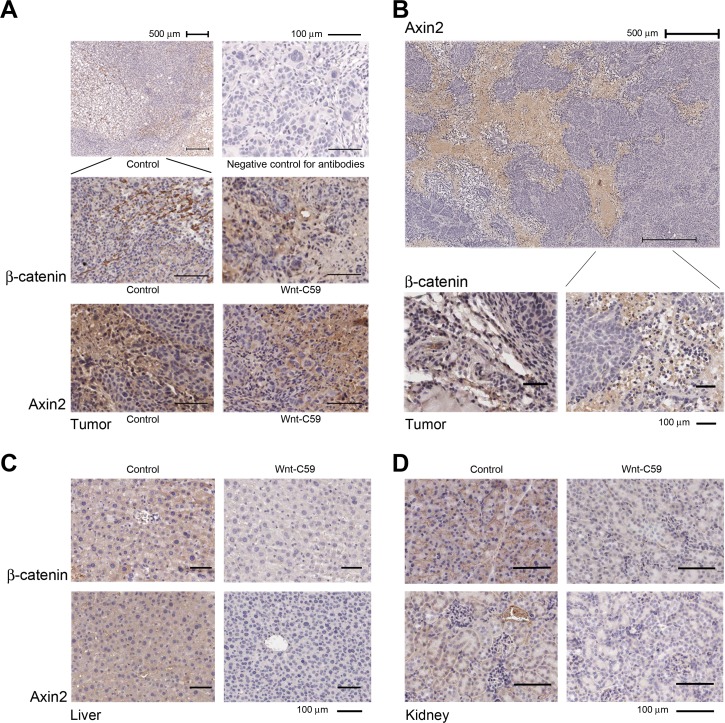
Wnt/β-catenin pathway is activated in the niche of growing tumors **A.** IHC staining shows that strong expression of both active β-catenin and Axin2 is detected in stromal cells around cancer nests derived from both control and Wnt-C59-treated mice with injected SUNE1 cells. The representative images of β-catenin expression under both higher and lower power objectives are presented, and a negative control without using primary antibodies is also included. **B.** Wnt signaling is up-regulated in tumor tissues obtained from control mouse injected with HNE1 cells and IHC staining shows that extensive expression of Axin2 is detected in stromal cells around cancer nests (top). The β-catenin is also expressed in stromal tissues of tumor (bottom). **C.** and **D.** Wnt-C59 has systemic effects in mice injected with HNE1 cells. Both active β-catenin and Axin2 expression are down-regulated in liver and kidney in Wnt-C59-treated mice, compared with those from control mice.

Wnt signaling in the stroma plays a vital role in the stem cell niche, as reported recently [24]. Therefore, we examined protein expression of active β-catenin and Axin2 in tumor tissues. These two proteins clearly accumulated in stromal cells around cancer nests, especially in the fast-growing region of the tumor tissues. No obvious difference of expression of these two proteins was detected between control and Wnt-C59-treated mice with injected SUNE1 cells, indicating that the tumor microenvironment in SUNE1 (Figure 4A) and HNE1 cells (Figure 4B) had favorable and up-regulated Wnt pathway activities. The Wnt-C59 had limited influence on these growing tumors in treated mice with injected SUNE1 cells.

Importantly, expression of active β-catenin and Axin2 in liver (Figure 4C) and kidney (Figure 4D) was clearly down-regulated in the Wnt-C59-treated HNE1 cell-injected mice compared to control mice. These findings confirmed that the administration of Wnt-C59 generated a systemic influence on the Wnt pathway in treated animals within a 41-day assay period, which induced tumor suppression in tested mice.

**Figure 5:**
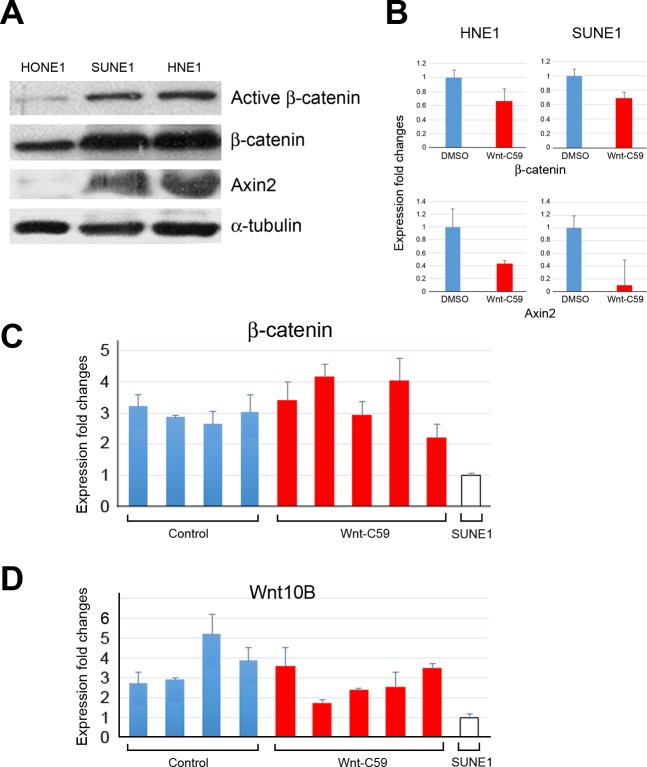
Wnt/β-catenin activities in HNE1 cells, SUNE1 cells, and tumor tissues **A**. Western blotting **reveals that Wnt/**β-catenin **signaling activities** in both SUNE1 and HNE1 cells are higher than that of control HONE1 cells, showing expression of active forms of β-catenin, and Axin2, a key regulator of the stability of β-catenin in the Wnt signaling in these two cell lines. **B.** qPCR analyses show the expression changes of Wnt pathway target genes following the treatment of Wnt-C59 in both HNE1 and SUNE1 cells. DMSO-treated sphere cells are used as controls. **C.** and **D.** qPCR analyses reveal expression changes of β-catenin and Wnt10B in tumor samples derived from both control and Wnt-C59-treated mice, compared to those of the parental cell line SUNE1 cells.

We recently reported that NPC HONE1 cells have very low endogenous activities of the Wnt pathway, especially expression of β-catenin [3]. Using this line as a control, we compared Wnt activities in HNE1 and SUNE1 cells. Western blotting confirmed that both HNE1 and SUNE1 cells expressed β-catenin and Axin2 proteins, which are key mediators of the Wnt pathway (Figure 5A). Notably, the active form of β-catenin was also detected in these two cell lines, reflecting that the activated Wnt/β-catenin signaling status in both HNE1 and SUNE1 cells, compared to control HONE1 cells.

Expression changes of β*-catenin* and *Axin2*, derived from Wnt-C59-treated and control spheres (DMSO) cells (Figure 3B), is seen in Figure 5B; expression of both genes was inhibited by Wnt-C59 (5 μM) in both HNE1 and SUNE1 cells.

To understand the influence of the Wnt pathway in tumor microenvironments, we examined SUNE1 cell-derived mouse tumors with and without Wnt-C59 treatment. Using parental SUNE1 cells as controls, the PCR assays showed the increased expression of β-catenin (Figure 5C) and Wnt10B (Figure 5D) in all tumor samples, including both control and Wnt-C59-treated mice. There is no obvious difference in the expression of β*-catenin* and *Wnt10B* between Wnt-C59-treated and control tumors, suggesting that Wnt pathway activities are similar in these tumors derived from SUNE1 cells. Because mouse tumors contain both SUNE1 and stromal cells, we compared β*-catenin* and *Wnt10B* expression to that of parental SUNE1 cells that had no stromal influences. As expected, mouse tumor tissues had higher Wnt activities compared to the *in vitro* cultured SUNE1 cell line. Additionally, Wnt10B expression, reported to be associated with Wnt-C59 treatment [7], increased with expression of β-catenin as expected. These results confirm the IHC staining findings detected in tumor tissues (Figures 4A and 4B), indicating that Wnt pathway activities, especially in the tumor microenvironment, stimulated SUNE1 cell tumor growth.

## DISCUSSION

A successful anticancer therapy should eliminate both the differentiated cancer cells and undifferentiated CSC populations. Although the CSC concept has been linked to resistance in cancer therapy for some tumor models, definitive evidence for functional suppression of tumor growth by manipulating both *Wnt* signaling and CSC activities is lacking. CSCs have the capacity to self-renew and this property is controlled by several developmental pathways, including *Wnt* signaling [3, 13, 28]. Although there is lack of direct evidence showing the existence of CSCs in unmanipulated solid tumors [2], many studies revealed the CSCs may be present in tumors as a distinct small population driving relapse and metastasis by giving rise to new chemoresistant tumors [1]. A hypothetical issue is whether Wnt inhibitors can inhibit CSC activities, since tumor stemness properties have long been suggested to correlate with both Wnt activation and tumor proliferation [1, 10, 29]. Therefore, it is of interest to understand whether both cancer stemness and proliferation activities can be altered by regulating the *Wnt* signaling cascade in tumor cells.

We focused on Wnt-mediated self-renewal signaling networks in NPC cells and detected that NPC stemness was precisely regulated by the physiological levels of *Wnt/*β*-catenin* signaling. We reported that multiple tumor suppressor pathways, including *p53* and *RB1*, within NPC cells are important for maintaining a balanced environment for upregulation of *β-catenin* expression [3, 30]. Additionally, we recently found that *BLU,* one of the TSGs mapped to the chromosome 3p21.3 region, controlled the NPC microenvironment and angiogenesis [31]. Since β-catenin is extensively activated in NPC tissues [32] and cell lines (seen in this study) and CSC properties are strongly regulated by microenvironment [4], it is possible that these TSGs play critical roles in both Wnt- or stemness-mediated activities in NPC. Given the facts that inflammation and Epstein-Barr Virus infection are very common in NPC cells [33], these microenvironment changes may also influence *Wnt* signaling and expression of TSGs through epigenetic regulation. Further elucidation of these interacting mechanisms underlying the Wnt pathway in NPC may provide useful insight for the development of an effective therapeutic approach.

One major stemness property in tumors is 3D sphere growth, which is widely used to explore dynamics, function, and regulation of stem cells for clonogenic growth potential [34]. To demonstrate the ability of Wnt inhibitors to suppress tumor stemness, we performed sphere inhibition assays in various NPC cells. In the ordinary culture conditions, these NPC cells usually grow as monolayers and do not form spheres. We now provide evidence confirming that cellular ability for 3D growth of some NPC cells is strongly correlated with the activation of the *Wnt*/β*-catenin* signaling and can be suppressed by the Wnt inhibitor, Wnt-C59.

NPC, like other human tumors, harbors genetically distinct subclones, which influence cellular heterogeneity in tumor behaviors. Recent study demonstrated that the *Wnt* pathway was responsible for this hierarchical configuration in tumor cells [35]. Like another *Wnt* inhibitor, ICG001, inhibitory effects of Wnt-C59 on cell proliferation and sphere formation were context-dependent in breast cancer [7]. However, the *PORCN* inhibitory approach is only suitable for a limited number of tumor cell lines having aberrant *Wnt* activation, as described previously. Our current investigations demonstrate that the great diversity of cells impacts tumor behavior and is an important and unavoidable issue faced when molecular therapy approaches are explored, as seen in CNE1, SUNE1, HNE1, and other HNSCC cells [36]. The *Wnt* pathway activities differ in these cells and influence responses to Wnt-C59 treatment. Consequently, the CNE1 stemness activity was not obviously inhibited by the *PORCN* inhibitor, reflecting heterogeneity of NPC cells and suggesting that other pathways, such as EGFR and AKT, possibly contribute to CNE1 stemness [37]. Although the *Wnt* pathway strongly correlates with stemness properties in some NPC cells, two Wnt inhibitors, ICG001 and XAV939, did not show obvious inhibitory effects on stemness in treated cells. These results suggest differences in regulatory networks are controlled by the complicated Wnt signaling in human tumor cells [8, 9].

We chose both SUNE1 and HNE1 cell lines with distinct growth dynamics for animal studies. The hypothesis is if Wnt signaling was dominant in the control of cellular stemness and proliferation, it would be possible to demonstrate the inhibitory effects by Wnt-C59 in these cell lines. Wnt-C59 treatment obviously delayed the growth of SUNE1 cells immediately after administration of tested chemicals in animals, but expression of active β-catenin protein was detected in tumor stroma. The findings suggested that Wnt-C59 was not able to fully control the CSC microenvironment to favor SUNE1-induced tumor growth in animals. It was also possible that some unknown negative feedback or redundant regulatory mechanisms finally dominated the signaling networks that caused restoration of Wnt activities and tumor cell proliferation in SUNE1 cells.

To detect long-term effects of Wnt-C59 in animals, which have not been reported yet, we investigated tumor growth of HNE1 cells. These cells require a longer latency period to form growing tumors in mice, reflecting greater intra-tumoral heterogeneity in this cell line. In the control group, surviving tumor cells, presumably CSCs, expanded in the injection sites and formed progressively growing tumors very quickly after the latency period. In contrast, injected tumor cells died and did not form visible and progressively growing tumors after the treatment of Wnt-C59 in animals. It was not possible to directly show the presence of CSCs in injected HNE1 cells, but the sphere inhibition assay demonstrates that Wnt-C59 could safely eliminate cells with stemness properties in an irreversible manner. Both *in vitro* and *in vivo* assays further confirm these findings and support the notion for the presence of CSCs in this cell line.

Targeting CSCs to suppress tumor growth is a major focus in current cancer research [38]. The current findings demonstrate that Wnt-C59 can effectively suppress *Wnt* signaling and stemness properties of certain tumor cells. We provide functional evidence demonstrating the use of an anti-CSC agent for tumor growth control is a feasible approach in cancer therapy and may be broadly exploited in human tumors.

## MATERIALS AND METHODS

### Cell lines and culture conditions

NPC CNE1, HK1, HNE1, HONE1, and SUNE1 cells were maintained in Dulbecco's modified eagle medium (DMEM) supplemented with 10% FBS. All these cell lines were obtained from the Hong Kong NPC AoE Cell Line Repository (Center for NPC Research at University of Hong Kong, Hong Kong) and have been authenticated by genotyping analysis. C666-1 cells were cultured in RPMI supplemented with 10% fetal bovine serum. Cells for sphere and sphere inhibition assays were cultured in DMEM/F-12 medium (Life Technologies, Grand Island, NY, USA) supplemented with 10 ng/ml epidermal growth factor (R&D Systems, Minneapolis, MN, USA), 10 ng/ml basic fibroblast growth factor (R&D Systems), and B-27 Supplement (Life Technologies). All tested cells for stemness were maintained in Nunc HydroCell 24 multidish (Themo Fisher Scientific, Roskilde, Denmark).

### Wnt inhibitors

Wnt-C59 was obtained from Cellagen Technology (San Diego, CA, USA) and was dissolved in DMSO making a 10 mM stock solution following the manufacturer's manual. Concentrations of 1 μM, 5 μM, 10 μM and 20 μM were used as working solutions in *in vitro* assays. ICG001 and XAV939 were obtained from R&D Systems and Cellagen Technology, respectively, and 10 mM stock solutions were prepared in DMSO. The concentrations of 5 μM and 10 μM of these two agents were used as working solutions for *in vitro* assays.

### Cell proliferation and sphere formation assays

Approximately, 1 × 10^4^ cells were seeded in 24-well plates, and Wnt-C59 (5 μM, 10 μM, and 20 μM) was added the next day. Each group was tested in triplicate and control groups with addition of DMSO were also established. Cell confluence was determined by microscopy at 24, 48, 72, and 96 hours after seeding of cells. The IC50 of Wnt-C59 was determined by MTT (3-[4,5-dimethylthiazol-2-yl]-2,5 diphenyl tetrazolium bromide) assay, using 96-well dishes. Next day, various concentrations of Wnt-C59 were added, and cellular viabilities were measured by a spectrophotometer at both 24 and 48 hours.

For sphere formation, approximately one hundred cells were seeded onto the Low Cell Bind Surface 24-well Nunc dish. Each group was done in triplicate and each well had 2 ml medium. Media were changed twice a week, and only half of the media was changed each time. Approximately, 1 × 10^3^ cells were seeded for each well in the sphere inhibition assay. At 1 to 5 days after plating, all tested cells formed small spheres. Five days later, Wnt-C59 (1 μM, 5 μM, and 20 μM) was added into experimental groups. Abilities for cell growth and sphere images were compared and recorded at the end of the first, second, and third weeks after addition of Wnt-C59, or DMSO in control groups. The sphere growths were observed and recorded daily under microscopy, and the area of spheres was analyzed using Metamorph (Molecular Devices, Sunnyvale, CA, USA) and recorded as average area (μm^2^).

### Western blotting

Western blotting was performed as previously described (3). The antibodies used in this assay are β-catenin (catalog # 9587, Cell Signaling,), Anti-Active β-catenin (catalog # 05-665, Millipore), and Axin2 (catalog # 2151, Cell Signaling). The α-tubulin (catalog # CP06, Calbiochem) was used as a loading control for all experiments.

### qPCR analyses

RNAs from tumor samples were extracted using the TRIzol reagent (Invitrogen, Carlsbad, CA, USA). Human GAPDH, primers GAAGGTGAAGGTCGGAGTC (forward) and GAAGATGGTGATGGGATTTC (reverse), was used as an internal control for all PCR reactions. The primers for qPCR are β-catenin: GGCTTGGAATGAGACTGCTGAT (forward) and CTGGCCATATCCACCAGAGTG (reverse); Axin2: CGCAGCAGTTTGGCGGCAGCA (forward) and AGGGTCCTGGGTGAACAGGTGGG (reverse); and Wnt10B: TGGGATGTGTAGCCTTCTCC (forward) and CCCAGCCAAAAGGAGTATGA (reverse). For qPCR analysis, triplicate PCR reactions were performed using the LightCycler 480 Real-Time PCR Instrument (Roche Diagnositics GmbH, Mannheim, Germany).

### Animal assay

The *in vivo* animal assays were performed as previously reported [39, 40]. A total of 1 × 10^7^ cells in 200 μl DMEM were injected into 4-8 week old nude mice subcutaneously. Next day, mice were randomly divided into two groups, experimental and control, and were given Wnt-C59 via vein injection. Based on previous reports [22-24], Wnt-C59 was dissolved in 30% propylene glycol for intravenous tail vein administration (2.5 mg/kg). After 48 hours, Wnt-C59 was added into drinking water (5 mg/kg/day) for experimental groups. Fresh water with Wnt-C59 was changed every 48 hours in dark bottles avoiding light; water consumption was recorded and calculated. Mice in control groups were treated with injection of 30% propylene glycol and given fresh water containing DMSO. Tumor sizes were measured three times a week until the last day of the experiment. Injected tumor cell nodule growth or shrinkage was recorded and only progressively growing tumors, confirmed by both gross and histology examinations, were counted. Tumor incidence was determined based on tumor numbers on the last day of experiment and termination day of assay was decided depending on possible appearance of ulcers on the surface of fast-growing tumors in control animals. All animal experiments were approved by the Government of Hong Kong Special Administrative Region and the University of Hong Kong.

### Histology and immunohistochemical staining

Mouse organs and tumor tissues were excised from mice, formalin-fixed, paraffin-embedded, and stained with H&E and IHC, as previously described (31). Primary antibodies of anti-Active β-catenin and Axin2 (Catalog # MABN259, Millipore) were used. The counterstain with hematoxylin was performed before mounting. The slides were scanned by ImageScope v11 software (Aperio, Vista, CA, USA).

### Statistical analysis

Differences between two groups were scored for statistical significances, using Student's t-test or Chi-square test.

## Abbreviations

NPC: nasopharyngeal carcinoma
CSCs: cancer stem cells
TSG: tumor suppressor gene
MBOAT: membrane bound O-acyltransferase
HNSCC: head and neck squamous cell carcinoma
3D: 3-dimensional.
